# Biomechanical Comparison of Fiber Tape Device Versus Transarticular Screws for Ligamentous Lisfranc Injury in a Cadaveric Model

**DOI:** 10.1177/03635465221118580

**Published:** 2022-08-22

**Authors:** Zachary A. Koroneos, Kristen M. Manto, Brandon J. Martinazzi, Chris Stauch, Shawn M. Bifano, Allen R. Kunselman, Gregory S. Lewis, Michael Aynardi

**Affiliations:** * Department for Orthopaedics and Rehabilitation, The Pennsylvania State University, Hershey, Pennsylvania, USA; † Division of Biostatistics and Bioinformatics, Department of Public Health Sciences, The Pennsylvania State University, Hershey, Pennsylvania, USA; Investigation performed at Penn State College of Medicine, Hershey, Pennsylvania, USA

**Keywords:** biomechanics of ligament, general sports trauma, foot, American football, dance, ballet

## Abstract

**Background:**

The preferred method of fixation and surgical treatment for ligamentous Lisfranc injuries is controversial. Transarticular screws, bridge plating, fusion, and flexible fixation have been described, yet none have demonstrated superiority. Furthermore, screw fixation and plating often require secondary surgery to remove implants, leading surgeons to seek alternative fixation methods.

**Purpose:**

To compare transarticular screws and a fiber tape construct under a spectrum of biomechanical loads by evaluating the diastasis at 3 joints in the Lisfranc complex.

**Study Design:**

Controlled laboratory study.

**Methods:**

Eight matched pairs of fresh, previously frozen lower extremity cadaveric specimens were fixed with either 2 cannulated transarticular crossed screws or a fiber tape construct with a supplemental intercuneiform limb. The diastasis between bones was measured at 3 midfoot joints in the Lisfranc complex: the Lisfranc articulation, the second tarsometatarsal joint, and the intercuneiform joint. Measurements were obtained for the preinjured, injured, and fixation conditions under static loading at 50% donor body weight. Specimens then underwent cyclic loading performed at 1 Hz and 100 cycles, based on 100-N stepwise increases in ground-reaction force from 100 to 2000 N, to simulate postoperative loading from the partial weightbearing stage to high-energy activities. Failure of fixation was defined as diastasis ≥2 mm at the Lisfranc articulation (second metatarsal–medial cuneiform joint).

**Results:**

There were no significant differences in diastasis detected at the Lisfranc articulation or the intercuneiform joint throughout all loading cycles between groups. All specimens endured loading up to 50% body weight + 1400 N. Up to and including this stage, there were 2 failures in the cannulated transarticular crossed-screw group and none in the fiber tape group.

**Conclusion:**

The fiber tape construct with a supplemental intercuneiform limb, which does not require later removal, may provide comparable biomechanical stability to cannulated transarticular crossed screws, even at higher loads.

**Clinical Relevance:**

Ligamentous Lisfranc injuries are common among athletes. Therefore, biomechanical evaluations are necessary to determine stable constructs that can limit the time to return to play. This study compares the biomechanical stability of 2 methods of fixation for ligamentous injury through a wide spectrum of loading, including those experienced by athletes.

The osseous and ligamentous arrangement in the midfoot constitutes the Lisfranc (tarsometatarsal) complex, providing stability to the archlike structure. The relatively limited stability of this region, lacking transverse stabilizers between the first and second metatarsals, is highly dependent on the oblique Lisfranc ligaments traversing from the medial cuneiform (MC) to the second metatarsal (2MT).^39,42,46^ The Lisfranc ligaments are composed of dorsal, plantar, and interosseous components in order of increasing strength based on previous biomechanical models.^4,15,35,46^

Isolated ligamentous Lisfranc injuries often occur because of trauma during sports or recreational activities.^41,46^ These ligamentous ruptures may present subtly without subluxation, dislocation, or fracture but will include pain, swelling, and tenderness over the area and are often misdiagnosed as a “foot sprain.” If left untreated, the ligamentous injury can lead to increased pain, instability, osteoarthritis, and morbidity.^4,18,46^ Furthermore, younger patients and athletes, eager to return to physical activity, may have prolonged periods of disability even with less severe ligamentous injuries.^11^

With recent systematic reviews showing opposing outcomes between primary arthrodesis and open reduction and internal fixation procedures, the controversy regarding ligamentous Lisfranc injury management continues.^2,19^ The goal of surgical intervention is primary reduction, with the most common technique utilizing cannulated transarticular crossed (CTC) screws. Multiple modes of fixation have been proposed as alternatives to CTC screws for open reduction and internal fixation procedures.

The purpose of this study was to compare 2 forms of internal fixation: (1) a fiber tape device passing intraosseously through the Lisfranc articulation with a dorsal intercuneiform **(IC)** adjunct vs (2) traditional CTC screws. We compared reduction and stability at the Lisfranc joint (MC-2MT) and the 2 adjacent joints (IC-2MT and IC-MC) after being exposed to a wide spectrum of loading, including that experienced during sports.

## Methods

### Specimen Selection and Preparation

**Figure 1.**
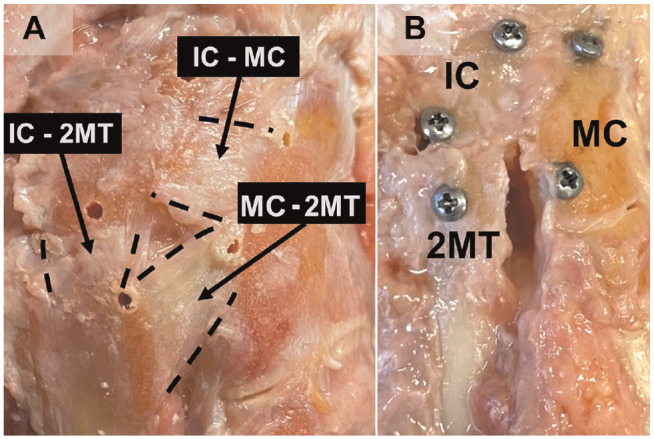
(A) The intact dorsal ligamentous arrangement displaying the 3 joints involved: IC-2MT (intermediate cuneiform–second metatarsal), IC-MC (intermediate cuneiform–medial cuneiform), and MC-2MT (medial cuneiform–second metatarsal). Holes in the bones are pilot holes for screw placement. (B) The isolated ligamentous Lisfranc injury at the MC-2MT joint with screw markers placed.

Eight matched fresh-frozen pairs of human cadaveric transtibial specimens (midtibia/fibula to toes; 16 specimens total) without preexisting foot deformity were obtained (Anatomy Gifts Registry) and prepared according to previous work.^7,24^ The cohorts consisted of 5 male and 3 female donors with an average age of 50 years (range, 25-68), an average height of 69 inches (range, 64-71), and an average weight of 158 lb (range, 115-260). All specimens were thawed overnight. The proximal tibia and fibula were dissected, and the subcutaneous fat and soft tissue were rigorously removed. The bones were dried with paper towels and sanded to create an adhering surface for the potting material. The tibia and fibula were fixed using syndesmotic screw fixation and potted in polyvinyl chloride piping using a urethane filler (Masters DYNA CAST; Free Manufacturing and Supply Co). The Achilles tendon and distal gastrocnemius muscle were isolated and kept intact during dissection and then whipstitched using a heavy Kevlar braided line for tensile loading (Emma Kites). Careful dissection involved the removal of the dorsal subcutaneous tissue while preserving the capsular attachments of the midfoot and maintaining the tie rod mechanism of the arch, which was performed according to previously published work.^21,24,38^ Miniature screws (2-mm head, 4-mm length), used as coordinate markers for diastasis measurements, were inserted into the 2MT, MC, and IC, which were predrilled with a Kirschner wire for pilot holes (Figure 1). Two screws were inserted within each bone and placed as close to the joints as possible without disrupting the path of operative repair to provide an accurate measurement of diastasis at each joint (Figure 1B).

### Ligamentous Lisfranc Injury Model

**Figure 2.**
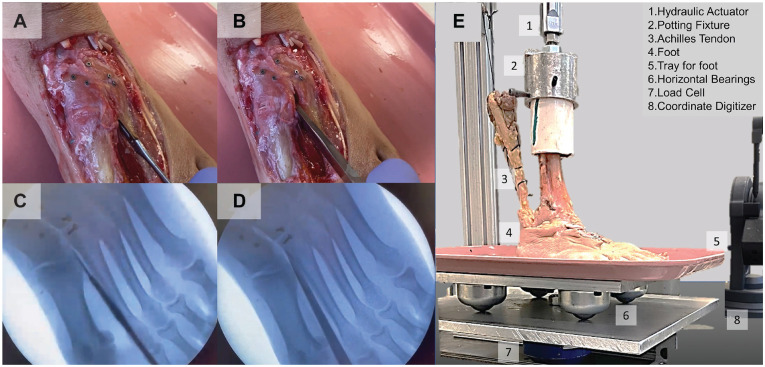
The creation of the purely ligamentous isolated Lisfranc injury using (A) a 10-blade scalpel to carefully section the dorsal, interosseous, and plantar Lisfranc ligaments and then (B) a narrow osteotome to widen the bones while under load. (C) The scalpel and (D) the osteotome were visualized under radiographic guidance to prevent disruption to any other ligamentous structure in the midfoot. (E) The loading apparatus with a numbered legend.

The purely ligamentous Lisfranc injury model was created using radiographic guidance in an apparatus that involved weighted plates as previously described.^24^ Specimens were placed under a static load equal to 50% of their donor body weight (BW) to simulate weightbearing radiographs, which are the most common and preferred method of identifying Lisfranc injuries (Figure 2, A-D).^39^ The dorsal, interosseous, and plantar Lisfranc ligaments were carefully sectioned using a 10-blade scalpel with no further disruption visualized at the more proximal intercuneiform joint as performed in previous work (Figure 2, A and C).^24^ Once sectioned, the joint space was widened using a narrow osteotome while avoiding injury to any other tarsal ligamentous structure (Figure 2, B and D). A successful injury model was defined as diastasis ≥2 mm observed at the Lisfranc articulation.

### Loading Apparatus

The polyvinyl chloride tube, encompassing the tibia and fibula, was fastened to the vertical actuator of a servohydraulic test frame (FlexTest 40; MTS), as performed in previous work.^5-7,24,37^ The braided line, whipstitched through the Achilles, was attached to a pulley mechanism of a pneumatic actuator to apply a tensile force.^24^ The foot rested on a plate supported by bearings that allowed translation and rotation in the horizontal plane perpendicular to the applied force. The ground-reaction force caused by the combined tibia and Achilles loading was measured with a load cell (1010ACK-500-B, eccentric load compensated; Interface) located below the plate that supported the bearings. Figure 2E presents an image of the loading setup.

### Surgical Repair

**Figure 3.**
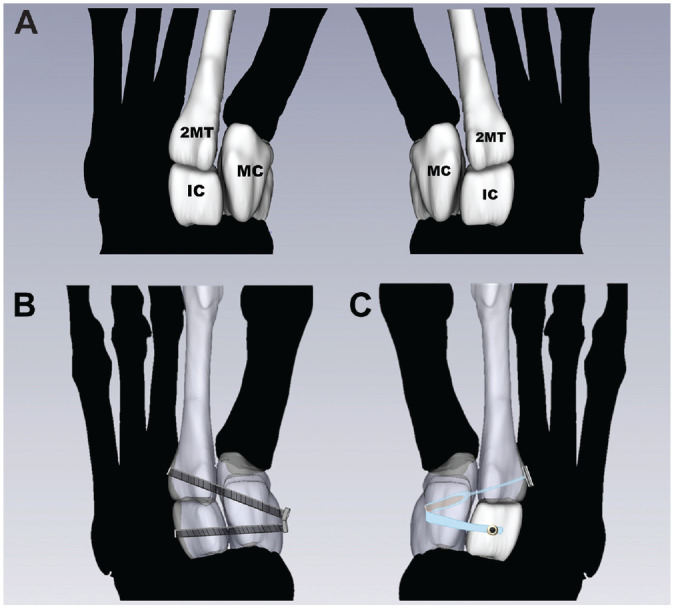
(A) Visualization of the native bones with gapping at the MC-2MT joint with labeled bones. The placement of repairs for (B) cannulated transarticular crossed screws and (C) the fiber tape device. 2MT, second metatarsal; IC, intermediate cuneiform; MC, medial cuneiform.

A linear bone clamp was used to reduce the Lisfranc diastasis, and the clamp was left in place during the surgical repair. Guide wires were utilized with fluoroscopy to ensure proper trajectory of the fiber tape device limbs and CTC screws for placement across the Lisfranc interval and the intercuneiform space. The 2MT-MC limb of the fiber tape repair (Arthrex Inc) utilized a 4.75-mm locking suture anchor in the medial aspect of the MC with an oblong button on the lateral side of the 2MT, while the supplemental limb was created using the same suture and a 3.5-mm anchor in the dorsal aspect of the IC (Figure 3C). The CTC screws (Arthrex Inc) were 4.0-mm titanium and driven interosseously through the articulations. The CTC screw trajectory was chosen in this study as it mirrors the path of fiber tape across Lisfranc articulations during repair (Figure 3). Successful reduction of the Lisfranc articulation was measured by fluoroscopy and digitized coordinates. After successful repair with either CTC screws or the fiber tape device, specimens were loaded. The fiber tape and screw fixation were performed on paired specimens with a single foot from each pair receiving one of the two fixations.

### Diastasis Measurements

Diastasis measurements for the 3 joints (2MT-MC, IC-MC, IC-2MT) were determined using a 3-dimensional (3D) coordinate digitizer (Microscribe G2 Series; Immersion Corp). Three-dimensional coordinate measurements were obtained for the aforementioned 2 dorsal screws in each bone crossing each joint (MC, IC, 2MT). The tip of the digitizer was inserted into the head of the screw, which helped to fix the axis of the stylus perpendicular to the top face of the screw. The diastasis for each stage of loading was calculated as the 3D Euclidean distance between 2 markers minus the distance measured at the state before cyclic loads were applied (“fixation”). The error of diastasis measurements was characterized by examining the absolute value of changes in intermarker distances between 2 markers in the same bone, which averaged 0.20 mm (SD, 0.19 mm; interquartile range, 0.25 mm).

### Experimental Protocol and Cyclic Loading

Diastasis measurements were longitudinally recorded at the following sequential conditions as in previous research: (1) “preinjury” with the native anatomy, (2) “injury” after Lisfranc ligament sectioning, (3) “fixation” with either the fiber tape device or CTC screws, and (4) after cyclic loading at the increments specified as follows.^24^ The diastasis was measured with the tibia statically compressed, resulting in ground-reaction force equal to 50% of the donor BW. The Achilles was loaded under tension with a force equal to 75% of this ground-reaction force load (37.5% BW).^12^

Cyclic loading, using 100-N stepwise increases in ground-reaction force, was performed similarly to previous work.^24^ Loading was performed at 100 cycles at 1 Hz and a peak magnitude of 50% BW + 100-N (compressive) ground-reaction force, followed by 100 cycles at 50% BW + 200 N, with the force increasing by 100 N every 100 cycles. For all cycles, the “valley” magnitude of load was 50% BW. Specimens were tested until the end of the “50% BW + 2000 N” cycles to evaluate the devices at a wide spectrum of loading that could simulate partial weightbearing to running.^33^ The Achilles tendon was held in constant tension at the aforementioned force. Three-dimensional coordinate measurements to evaluate the diastases, as described earlier, were taken after every 200 cycles. The diastasis measurements were made in the following order: proximal 2MT, distal 2MT, distal MC, proximal MC, proximal IC, and distal IC. Measurements were made during a 60-second dwell period immediately after the last cycle every 200 N.

Diastasis at the Lisfranc joint (MC-2MT) ≥2 mm was indicative of failure based on previous studies evaluating this structure and other ligaments in the foot.^7,14,34,48^

### Statistical Analysis

Linear mixed-effects models were used to compare the diastasis measurements for each fixation type (CTC screws vs fiber tape device with supplemental limb) for each of the 3 measured joints at each interval of cyclic loading.^16,24^ A random specimen donor effect accounted for 2 repeated factors per donor: method of fixation and peak force during cycles. The method of Kenward and Roger^23^ was utilized to determine the denominator degrees of freedom, and the estimation method used within the linear mixed-effect models was the restricted maximum likelihood. The set inequality for statistical significance was *P* < .05. Analyses were performed in SAS software Version 9.4 (SAS Institute Inc), and all hypotheses were tested as 2-sided. In addition, the censored paired data were compared using a paired Prentice-Wilcoxon test.^47^

## Results

### Validating the Injury Model and Fixation

The injury model created diastasis mean ± SE values of 2.71 ± 0.44 mm (range, 2.13 to 3.73), 0.27 ± 0.56 mm (−0.56 to 0.68), and −0.05 ± 0.44 mm (−0.68 to 0.88) for the MC-2MT, IC-MC, and IC-2MT joints, respectively, across all specimens from all groups before fixation, and there were no significant differences between fixation groups (MC-2MT, *P* = .55; IC-MC, *P* = .41; IC-2MT, *P* = .17). The fiber tape device group had an average MC-2MT diastasis decrease of −2.81 ± 0.52 mm (2.44 to 3.71) while the CTC screw group had an average decrease of −2.61 ± 0.32 mm (2.23 to 3.17), which was nonsignificant between groups (*P* = .34).

### Cyclic Loading and Diastasis Analysis

**Figure 4.**
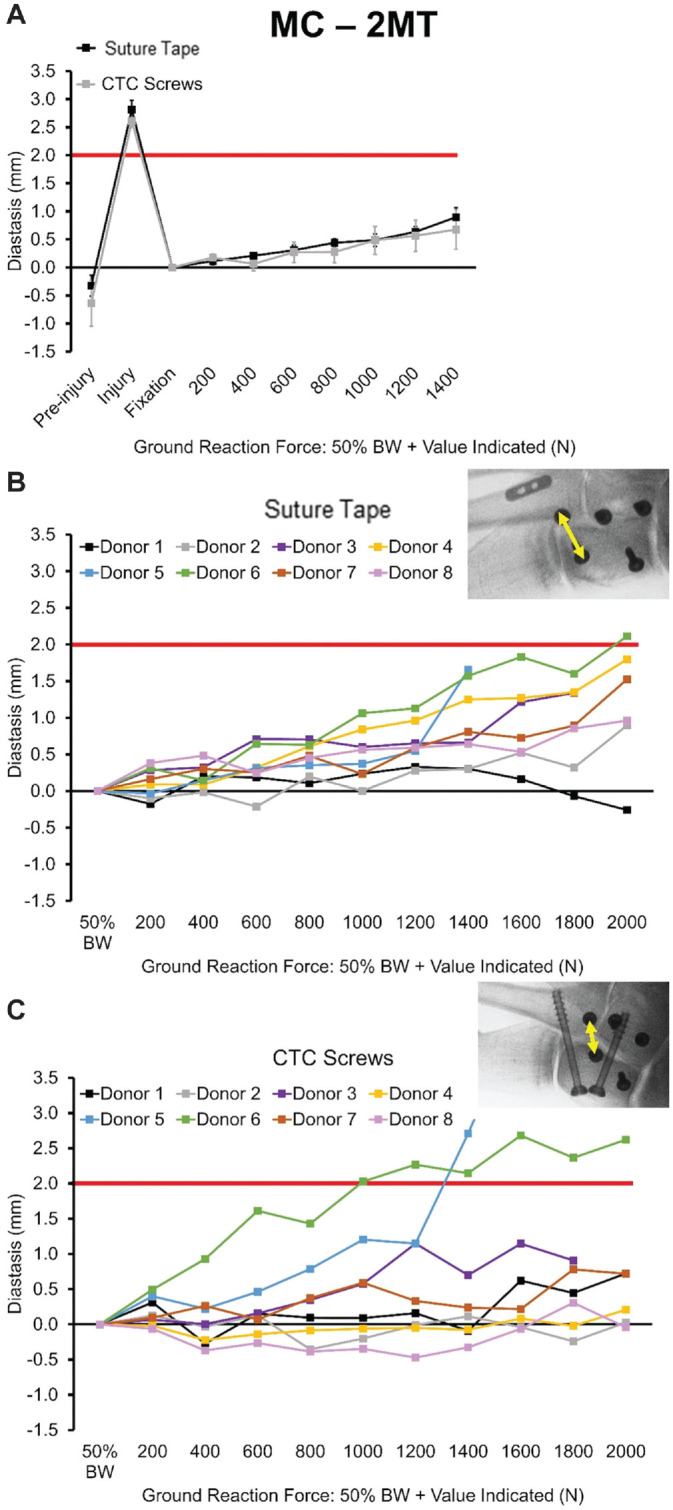
Diastasis values at the Lisfranc joint (MC-2MT) for the fiber tape device and CTC screws. Change in 3D distance is indicated by arrows. (A) Mean ± SE values up to 50% BW + 1400-N ground-reaction force. Results for individual paired specimens are shown for (B) the fiber tape device group and (C) the CTC screw group. Inset image shows 3D distance. 3D, 3-dimensional; BW, body weight; CTC, cannulated transarticular crossed; MC-2MT, medial cuneiform–second metatarsal.

**Figure 5.**
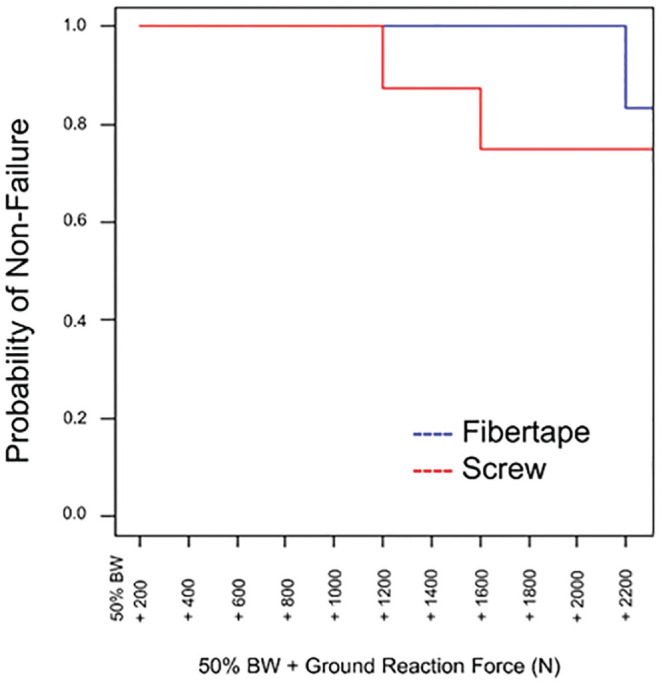
Kaplan-Meier plot displays the probability of nonfailure for each fixation type at each loading stage. Probability is shown as a ratio of the specimens that did not reach ≥2 mm of diastasis at the medial cuneiform–second metatarsal joint. BW, body weight.

The MC-2MT diastases with respect to cyclic loading stage are depicted in Figure 4. All specimens endured loading without the failure of secondary stabilizers up to at least 50% BW + 1400 N (1400 cycles) (Figure 4A). Screws and fiber tape were still intact after all loading. The pooled variability of 0.35 mm that we observed, our study had >80% power to detect a minimal difference of 0.41 mm. There were no significant differences detected at any stage of cyclic loading between groups at the MC-2MT Lisfranc joint. The mean ± SE MC-2MT diastasis through 50% BW + 1400 N was 0.90 ± 0.50 mm (range, 0.30 to 1.65) for the fiber tape group and 0.68 ± 1.06 mm (−0.32 to 2.71) for the CTC screw group. Between this loading stage and 50% BW + 2000 N, testing of 2 pairs of specimens (donors 3 and 5) was halted during loading owing to instability associated with excessive abduction. At 50% BW + 2000 N, 6 matched pairs of specimens remained, with diastasis averages of 1.17 ± 0.77 mm (−0.25 to 2.11) and 0.71 ± 0.91 mm (−0.04 to 2.62) for the fiber tape and CTC screw groups, respectively. By this loading stage, 1 specimen in the fiber tape group reached the failure threshold, although a second specimen from this group (donor 5) exceeded 1.5-mm diastasis earlier in loading before testing was halted for loading control reasons (Figure 4B). The paired Prentice-Wilcoxon test, used to compare the censored paired data, also showed no significant differences between groups. Figure 5 illustrates the probability of nonfailure in a Kaplan-Meier plot.

**Figure 6.**
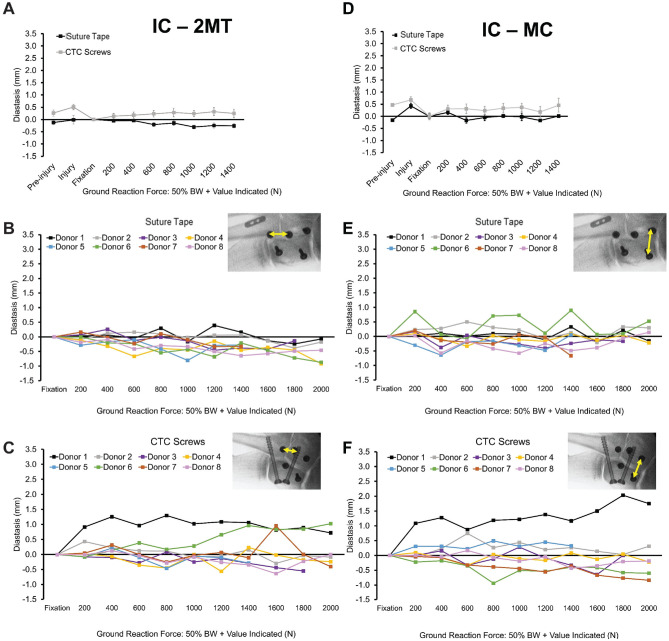
Diastasis values at (A-C) the second tarsometatarsal joint (IC-2MT) and (D-F) the intercuneiform joint (IC-MC) for the fiber tape device and CTC screws. (A, D) Mean ± SE values up to 1400-N ground-reaction force. Results for individual paired specimens are shown for (B, E) the fiber tape device group and (C, F) the CTC screw group. Inset image shows 3-dimensional distance. BW, body weight; CTC, cannulated transarticular crossed; IC-2MT, intercuneiform–second metatarsal; IC-MC, intercuneiform–medial cuneiform.

The IC-2MT and IC-MC joint diastases are shown in Figure 6. The fiber tape group had significantly less IC-2MT diastasis than the CTC screw group at 50% BW + 1000 N (*P* = .030), 50% BW + 1400 N (*P* = .022), and all subsequent stages through 50% BW + 2000 N (*P* < .024) (Figure 6, A-C). Considering all 8 pairs at 50% BW + 1400 N, the average diastases were −0.26 ± 0.34 mm and 0.17 ± 0.56 mm for the fiber tape and CTC screw groups, respectively. At 50% BW + 2000 N, the average diastasis was −0.50 ± 0.39 mm for the fiber tape group and 0.20 ± 0.63 mm for the CTC screw group.

At the intercuneiform joint (IC-MC), the average diastasis was 0.01 ± 0.49 mm for the fiber tape group and 0.04 ± 0.55 mm for the CTC screw group at 50% BW + 1400 N including all 8 pairs (Figure 6D). At 50% BW + 2000 N, the average diastases were 0.12 ± 0.31 mm and 0.06 ± 0.85 mm for the fiber tape and CTC screw groups, respectively, with 6 matched pairs remaining (Figure 6, E and F). There were no statistically significant differences detected at this joint at any stage of loading.

## Discussion

The MC-2MT Lisfranc joint and IC-MC diastases showed no significant differences between the fixation groups. Up to and including this stage, there were 2 failures (≥2-mm MC-2MT diastasis) in the CTC screw group and none in the fiber tape group (Figure 4, B and C). There was significantly less diastasis at the IC-2MT joint in the fiber tape group when compared with CTC screws in loading stage 50% BW + 1400 N and greater. However, the detected diastases were relatively low at this joint (<0.60 mm).

CTC screw fixation is the most common repair of ligamentous Lisfranc injuries to maintain rigid fixation after reduction.^§^ Yet, disadvantages including damage to the articular surfaces of joints, retained intra-articular threads, and the need for additional surgery to remove hardware have led surgeons to seek alternatives.^1^ Flexible fixation devices have recently been proposed as alternatives, and a fiber tape device with a supplemental intercuneiform limb attached to the dorsal aspect of the IC maintained reduction at high loads.^24^ On the basis of these data, we sought to compare the commonly performed CTC screw technique with what we believe is the most relevant and adequate flexible fixation. To our knowledge, this is the first cadaveric biomechanical study comparing a fiber tape device with a supplemental intercuneiform limb to CTC screws. Additionally, no study of the Lisfranc complex has evaluated the joint spaces at loads based on the BWs of donors to simulate diastasis during a weightbearing radiograph or computed tomography. Finally, the model utilized is only the second to increase the ground-reaction force up to loads that simulate those experienced by active patients and athletes, greatly exceeding that applied in most biomechanical models of this injury.^11,17,24,31,32^

The data demonstrate that fiber tape and CTC screws provide biomechanical stability in repairing ligamentous Lisfranc injuries while generally withstanding high loads (Figure 4). Three specimens reached ≥2 mm of diastasis at the MC-2MT joint, which was indicative of failure, with 1 being in the fiber tape group and 2 in the CTC screw group. All specimens failed at loads >1330 N. The results of this study also suggest that the use of a fiber tape device with a dorsally placed supplemental intercuneiform limb tended to provide more flexible fixation allowing for some joint motion (Figure 6B). The 6 specimens in the fiber tape group that reached 1 mm of diastasis at the MC-2MT joint did so at an average ground-reaction force of 289% ± 98% donor BW, whereas 3 CTC screw specimens reached 1 mm at 207% ± 49% donor BW. There were no significant differences in diastasis at the Lisfranc joint (MC-2MT) and at the intercuneiform joint (IC-MC). While diastases remained relatively low in both groups at the IC-2MT joint, there was a statistically significant difference between groups. At this joint, the fiber tape group displayed a decrease in diastasis over the duration of loading while the CTC screw group had an increase (Figure 5, B and C).

While elongation is the most intuitive cause of diastasis in the fiber tape group, the reason behind the CTC screw diastasis is less clear. Modes of screw fixation include screw cutout or loosening, screw fracture, and bony fracture. As no implant failures and no gross bony fractures were observed, a plausible cause may be the screws cutting interosseously through the bones. In addition, the 2 specimen failures within the CTC screw group were both female (ages, 39 and 68 years).

The injury model in this study involved sectioning all 3 components (dorsal, interosseous, plantar) of the Lisfranc articulation to yield the most instability from the isolated injury. An advantage of this injury model was that the ligaments were sectioned under 50% BW loading that did not overconstrain the foot and could reproduce at least 2 mm of diastasis at the MC-2MT joint for all specimens.^32,35^ By performing this careful sectioning under fluoroscopy in the anteroposterior plane, we ensured that the intercuneiform joint was not disrupted. Furthermore, the injury model was confirmed through 3D coordinate measurements displaying relatively low diastasis at the IC-MC and IC-2MT joints after sectioning.

There have been 2 studies to date that have biomechanically compared screws with flexible fixation devices for Lisfranc injuries. Panchbhavi et al^36^ compared a single screw and a suture button, without intercuneiform stabilization in either procedure. Nery et al^32^ compared CTC screws and a fiber tape device, also without a supplemental intercuneiform limb. Both these studies reported no significant differences between the flexible fixation methods and their comparator screws. Unlike in our study, both these studies utilized a biomechanical model that constrained the ankle and the forefoot and loaded specimens to a maximum axial load of 343 and 400 N. In a separate recent study of fiber tape reconstructions from our laboratory, we determined significantly less MC-2MT diastasis at higher loads for the fiber tape device with a supplemental intercuneiform limb, when compared with fiber tape alone, under similar loading conditions to those tested in this study.^24^

While ligamentous evaluation through 3D diastasis has become a standard approach, controversy exists over the exact mechanism of injury and ideal biomechanical models to test Lisfranc reconstruction.^1,15,32,35,45^ Some studies have loaded the foot in a fixed plantarflexed position based on reports of this being a common injury mechanism.^3,8,26,28,35,38,39^ These models, which involve “locking” the foot in place through the ankle using Steinman pins or screws, assess the Lisfranc joint with applied axial loads as low as 100 N and a maximum of 400 N.^1,8,26,28,38,45^ The plantarflexed position may increase shear force between bones and bending moment at the location of the Lisfranc complex; however, the loads tested represent only those experienced during partial weightbearing.^33,40^ This is likely due to inherent instability in this setup, which we observed in our pilot testing. A recent study evaluating different screws potted the hindfoot, which allowed for static loads up to 1100 N to be tested.^17^ The present biomechanical model allowed for external rotation and pronation without constraint, with the use of the bearing plate.^24^

Previous literature stated that the position with the highest percentage of Lisfranc injuries in football players is offensive lineman.^29^ Ten years of National Football League (NFL) Combine data indicated that the average NFL offensive lineman weight was approximately 315 lb (~1400 N).^4^ In the stance phase of gait alone, which produces loads slightly larger than full BW, this would provide much larger stresses than most studies, which tested between 250 and 400 N. In addition, a video analysis study involving Lisfranc injuries in NFL players determined that 90% occurred while engaging another player, further increasing these loads.^22^ Loads that greatly exceed those involved in gait are required to simulate these types of instances for athletes.

Studies evaluating postoperative protocols for professional athletes have recommended 6 to 8 months before engaging in limited activities, when hardware removal is necessary, and current management outcomes have shown that return to play is nearly a year for Lisfranc injuries.^20,27^ While both fixations withstood relatively high loads, the fiber tape device does not require the planned second surgery, which may allow for faster return to play. By way of a cadaveric model being used in the present study, there is no healing present, nor can healing time be evaluated. However, this study does support the need for a clinical prospective study as the loads tested may provide some evidence for allowing earlier weightbearing and recovery. Although we believe that this study may set the framework for a clinical outcome study, the purpose of the present study was to evaluate biomechanical efficacy and safety.

The main limitation of this study is that measurements were obtained for only the dorsal aspect of the joint. Moreover, after each interval of cyclic loading, measurements were obtained at a static load of 50% BW rather than at the peak loading force (which is a common approach in biomechanical studies). These measurements were obtained immediately after the cessation of cyclic loading in the same order of markers for all specimens to reduce the variability in creep; yet, the direct effects of creep were not analyzed. Given that measurements were obtained in the same order and within a relatively small time window, we expect that there could be small systematic effects of creep among the various diastasis outcomes. Nevertheless, since we had matched pairs, any creep effects would be similar between treatment groups. Specimens were loaded consistently for approximately 1 hour, and we obtained measurements at a static load that was less than the peak loads during our cyclic loading. The cyclic loading can be viewed partly as a preconditioning that should reduce the effects of creep during measurements. Additionally, measurements at loads <800 N, for the reason of smaller loading magnitude, could have been more influenced by system errors. Additionally, the range of donor ages may provide differences in bone and tissue quality, although this issue is often encountered in cadaveric studies. The simplification of loading and standardized failure criteria were also limitations; however, these methods have been previously published.^7,24^ A limitation of our study attributed to budgetary restrictions was use of 8 matched pairs of lower extremity cadaveric specimens, similar to other studies.^36,38^

## Conclusion

In this cadaveric biomechanical study, the use of CTC screws or a fiber tape device with a supplemental intercuneiform augmentation appeared to provide similar biomechanical stability. The fiber tape device may serve as a viable alternative to screw fixation, while obviating the need for hardware removal. Future work should evaluate clinical outcomes of patients, especially in regard to their rehabilitation protocols and potentially with the use of weightbearing computed tomography scans.

## Footnotes

§ References 1, 2, 9, 10, 13, 25, 30, 32, 43, 44.
